# Helminths of urban rats in developed countries: a systematic review to identify research gaps

**DOI:** 10.1007/s00436-020-06776-3

**Published:** 2020-06-30

**Authors:** Diana S. Gliga, Benoît Pisanu, Chris Walzer, Amélie Desvars-Larrive

**Affiliations:** 1 Conservation Medicine, Research Institute of Wildlife Ecology, University of Veterinary Medicine, Vienna, Austria; 2 Unité Mixte de Services (UMS) 2006 Patrimoine Naturel, Office Français pour la Biodiversité (OFB), Muséum National d’Histoire Naturelle (MNHN), Paris, France; 3 Health Program, Wildlife Conservation Society, Bronx, NY USA; 4 Unit of Veterinary Public Health and Epidemiology, Institute of Food Safety, Food Technology and Veterinary Public Health, University of Veterinary Medicine, Vienna, Austria; 5 Complexity Science Hub, Vienna, Austria

**Keywords:** *Rattus rattus*, *Rattus norvegicus*, Helminth, Urban habitat, City, Review

## Abstract

Although black (*Rattus rattus)* and brown (*Rattus norvegicus*) rats are among the most widespread synanthropic wild rodents, there is a surprising scarcity of knowledge about their ecology in the urban ecosystem. In particular, relatively few studies have investigated their helminth species diversity in such habitat. We followed the PRISMA (Preferred Reporting Items for Systematic Reviews and Meta-Analysis) guideline to synthesize the existing published literature regarding the helminth fauna of urban rats in developed countries (North America, Europe, Australia, New Zealand and Japan). We aimed at describing the species diversity and richness of urban rat helminths, the species prevalence and associations, the methods of investigation, the pathological changes observed in the hosts, the risk factors of infection and the public health significance of rat-borne helminthiases. Twenty-three scientific papers published between 1946 and 2019 were reviewed, half of them were conducted in Europe. Twenty-five helminth species and eight genera were described from the liver, digestive tract, lungs and muscles of urban rats. The most commonly reported parasite was *Calodium hepaticum*. Prevalence and risk factors of helminth infection in urban rats varied greatly between studies. Observed pathological findings in the rat host were generally minor, except for *C. hepaticum*. Several rat helminths can parasitize humans and are therefore of public health significance. The lack of references to identification keys and the rare use of molecular tools for species confirmation represent the main limitation of these studies. Knowledge gap on this topic and the needs for future research are discussed.

**Electronic supplementary material:**

The online version of this article (10.1007/s00436-020-06776-3) contains supplementary material, which is available to authorized users.

## Introduction

The urbanisation process, together with the climate change and increasing habitat loss and pollution, alters the distribution range, physiology, biology and behaviour of many wildlife species (Prange et al. 2003; Partecke et al. 2006; French et al. 2008; McKinney 2008; Costantini et al. 2014; Meillère et al. 2015). Within the fragmented urban ecosystem, modified habitat structures, increased resource availability, reduced biodiversity and altered trophic interactions affect wildlife population biology, hence the nature and functioning of wildlife communities (Bradley and Altizer 2007; Cavia et al. 2009; Horsák et al. 2013; Aronson et al. 2016). These changes are subsequently affecting host-parasites interactions, parasite transmission and the structure of the parasite communities (Bradley and Altizer 2007; Cable et al. 2017), potentially leading to an augmented risk of spillover of wildlife parasites to humans (Gordon et al. 2016).

Rats (*Rattus* spp.) benefit from urban sprawl (McKinney 2006) and, over the past centuries, they have become one of the most successful urban wildlife species. Despite their close proximity with humans, the biology and ecology of urban rats have been understudied (Himsworth et al. 2013). If bacterial and viral organisms carried by rats know nowadays an increasing scientific interest (particularly if they are zoonotic) (Himsworth et al. 2013; Desvars-Larrive et al. 2017; Angley et al. 2018; Strand and Lundkvist 2019), studies on the helminth fauna of urban rats remain scarce. Helminths can modify wildlife host population dynamics (Hudson et al. 1998; Albon et al. 2002), communities (Mouritsen and Poulin 2002) and health status (Tompkins et al. 2011). Therefore, knowledge on urban rat helminths could interest (urban) ecologists but also pest management professionals. Moreover, several helminths carried by urban rats are zoonotic (Meerburg et al. 2009; Gordon et al. 2016). Rodent-borne helminthiases are neglected diseases that mostly (but not exclusively) affect people from developing, low-income countries (World Health Organization 2012). They can impact human nutritional status, inducing negative effects at different stages of the human life cycle (Crompton and Nesheim 2002), exacerbate malaria and HIV/AIDS, impair vaccine efficacy (World Health Organization 2012) and enhance the risk of allergy (Sitcharungsi and Sirivichayakul 2013).

This systematic review aimed at compiling and summarizing the peer-reviewed literature on the helminths of urban rats, i.e. the brown rat, *Rattus norvegicus* (Berkenhout, 1769), and the black rat, *Rattus rattus* (Linnaeus, 1758). Helminths require relatively specific biotic and abiotic conditions to complete their life cycle (Froeschke and Matthee 2014), therefore, we restricted the scope of this systematic review to urban ecosystems within the so-called developed regions, as defined by the United Nations (i.e. North America, Europe, Australia, New Zealand and Japan) (United Nations 2016). Indeed, urban habitats in developed countries present a common general framework (Johnson and Munshi-South 2017) that includes relatively similar sanitary (e.g. water quality) and climatic conditions, and provides comparable environmental situations to the parasites and their hosts.

In this systematic review, we addressed the following questions:

1. Which helminth species infect urban rats and what are their prevalence?
2. What are the methods used to investigate the helminths of urban rats?
3. What are the risk factors for infection in the rat host?
4. What are the pathological findings related to helminth infection in the rat host?
5. What is the veterinary and public health importance of rat-borne helminthiases in cities?

## Materials and methods

### Search strategy

We performed a systematic search following the PRISMA (Preferred Reporting Items for Systematic Reviews and Meta-Analyses) guideline for systematic reviews (Moher et al. 2009). Our search included the databases CAB Direct, JSTOR, Pubmed, Web of Science Core Collection and Scopus. We searched the databases between inception and the cut-off date 23 March 2019. The literature was searched using the following query:

(((“*Rattus norvegicus*” OR “Norway rat*” OR “brown rat*” OR “*Rattus rattus*” OR “black rat*” OR “roof rat*”) AND (worm* OR parasite* OR helminth* OR nematode* OR cestode* OR round worm* OR tapeworm*)) AND (urban* OR cities OR city OR municipal* OR suburban OR residential OR metropol*)).

### Study selection

References identified by the search were screened for inclusion criteria and relevance to the review question by two of the co-authors (ADL and DG). Discrepancies were resolved by consensus. Studies were selected using the following inclusion criteria: studies published from inception to 23 March 2019, studies in English language, studies published in peer-reviewed journals, studies that investigated wild *R. rattus* and/or *R. norvegicus* as hosts, studies that investigated helminth parasites, studies conducted in urban ecosystems and studies conducted in developed countries (United Nations 2016). A major challenge in urban ecology is to clearly delineate the urban ecosystem (McIntyre et al. 2000). Regional- or country-scale differences in urban planning make it difficult to find criteria that adequately define what is “urban” at a global scale (United Nations 2018). Due to this heterogeneity, we relied on the categorisation of the study sites by the authors of the publications when determining their eligibility for our systematic review

Non-English studies, as well as grey literature, reviews, books, book chapters, conference papers and poster abstracts, were excluded. Furthermore, we did not consider studies investigating other species than *R. rattus* or *R. norvegicus*, studies conducted in rural ecosystems or unspecified locations, experimental studies and studies conducted on captive animals.

### Data extraction and synthesis

For each study, two co-authors (ADL and DG) extracted the relevant data organized in 22 categories (Table 1) and compiled them into a database. When necessary, appendices and supplementary materials were also inspected. For each helminth species, we recorded the Latin binomial (i.e. the scientific name) and its synonyms as quoted in the article. Maps were built using QGIS 3.4.5 (QGIS Development Team 2018). The R package *ggplot2* (Wickham 2016) was used for data visualisation.

**Table 1:** Data extracted from the selected publications and used to address the review questions

Category	Definition
Year	Year of publication
Reference	Authors. Year. Title. Journal. Volume. Issue. Pages
Research area	Journal scope
Scale of the study	Spatial scale: a city block, one city, multiple cities, a country
Location	Location(s) of the study
Trapping site(s)	Description of the trapping site(s)
Method of capture	Type(s) of trap
Host species	*Rattus* species investigated: *R. rattus* and/or *R. norvegicus*
Aim of the study	Main objective(s) of the research
Global relevance	Main scientific field covered by the study
Method(s) used for detection	Method(s) used to retrieve the helminths or evaluate intensity of infection
Morphological identification keys	Reference paper(s) cited for the identification of the helminth species
Sample size	Number of rats investigated
Helminth species	Latin binomial as quoted in the article
Organ	Organ where the helminth species was retrieved
Number of positive	Number of positive rats for each helminth species
Prevalence	Prevalence of infection for each helminth species (number of positive / sample size)
Helminth species richness	Number of helminth species per host (co-infection)
Parasite burden	Defined using different metrics: mean intensity (total number of helminths of a particular species found in a sample divided by the number of hosts infected with that helminth); abundance (number of individuals of a particular helminth species in a single host); mean abundance (total number of individuals of a particular helminth species in a sample of a particular host species divided by the total number of hosts of that species examined (including both infected and uninfected hosts), i.e. the average abundance of a helminth species among all members of a particular host population) (Bush et al. 1997)
Pathological findings	Gross and histological changes induced by the presence of an helminth species
Interspecies interaction	When the occurrence of one helminth species has an impact on the presence of another species
Risk factor(s) of infection	Risk factor(s) of infection statistically identified

## Results

### PRISMA-guided study selection

**Fig. 1:**
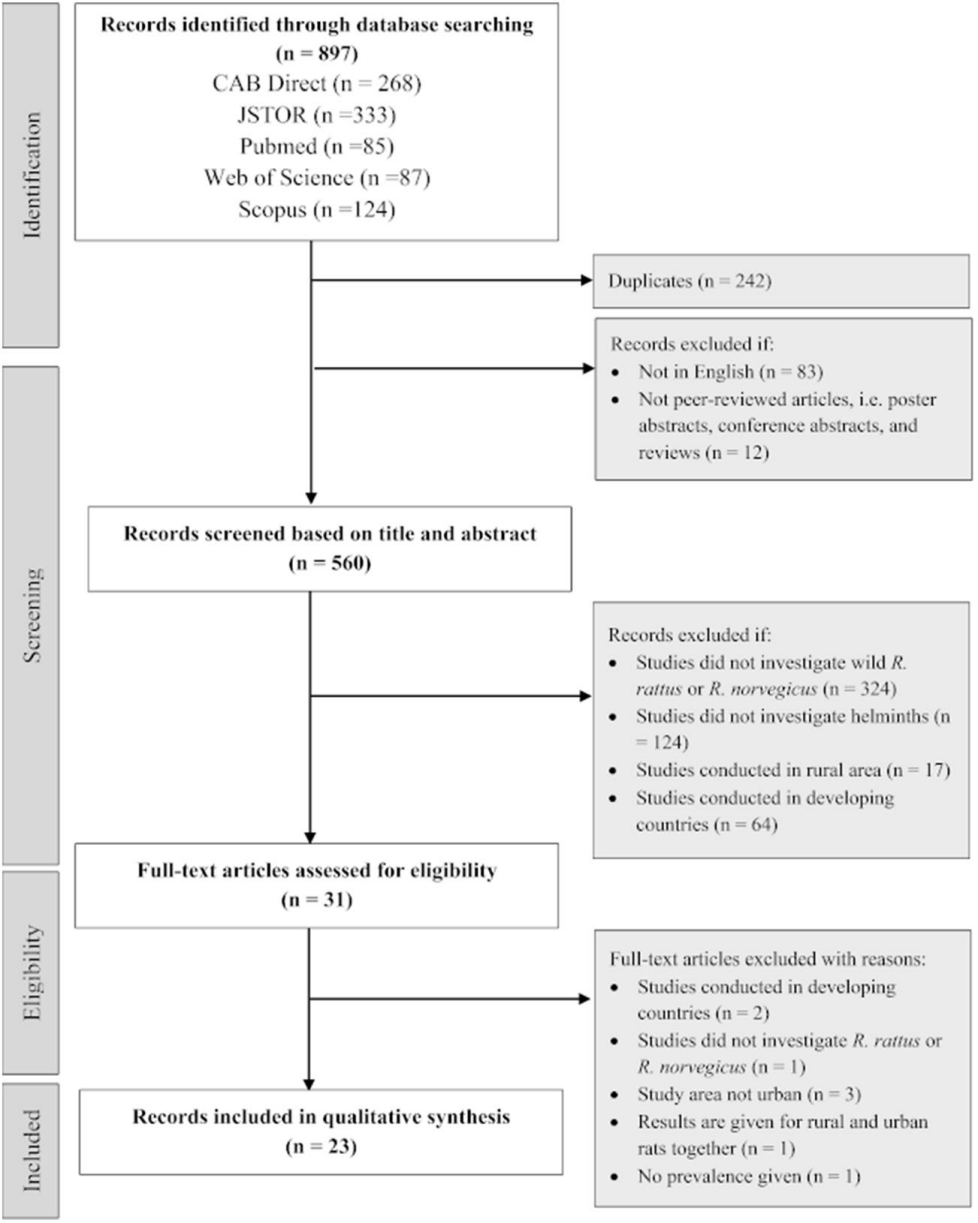
PRISMA flow diagram of the methodology and selection process

The flow diagram of the search strategy steps is presented in Fig. 1. Our first search yielded 897 results (268 from CAB Direct, 333 from JSTOR, 85 from Pubmed, 87 from Web of Science, and 124 from Scopus). After exclusion of duplicates (*n* = 242), reviews, poster presentations, proceeding papers (*n* = 12), and non-English publications (*n* = 83), 560 records were retained for the screening of titles and abstracts. Articles were excluded if the studies did not investigate wild *R. rattus* or *R. norvegicus* (*n* = 324), did not investigate helminths (*n* = 124), was conducted in rural area (*n* = 17) and/or in developing countries (*n* = 64). Thirty-one full-text articles met the eligibility criteria. After full examination, two of them were removed because they were not conducted in developed countries, one was excluded because it did not investigate *R. rattus* or *R. norvegicus*, three were excluded because they were not conducted in urban settings and two were excluded because prevalence could not be extracted from the results. Many studies had to be excluded on the basis of more than one criterion; numbers given in Fig. 1 indicate the first criterion of exclusion that has been identified. A total of 23 studies were ultimately included in our review. The details of the included studies are shown in the Supplementary Material 1.

### Types of studies

**Fig. 2:**
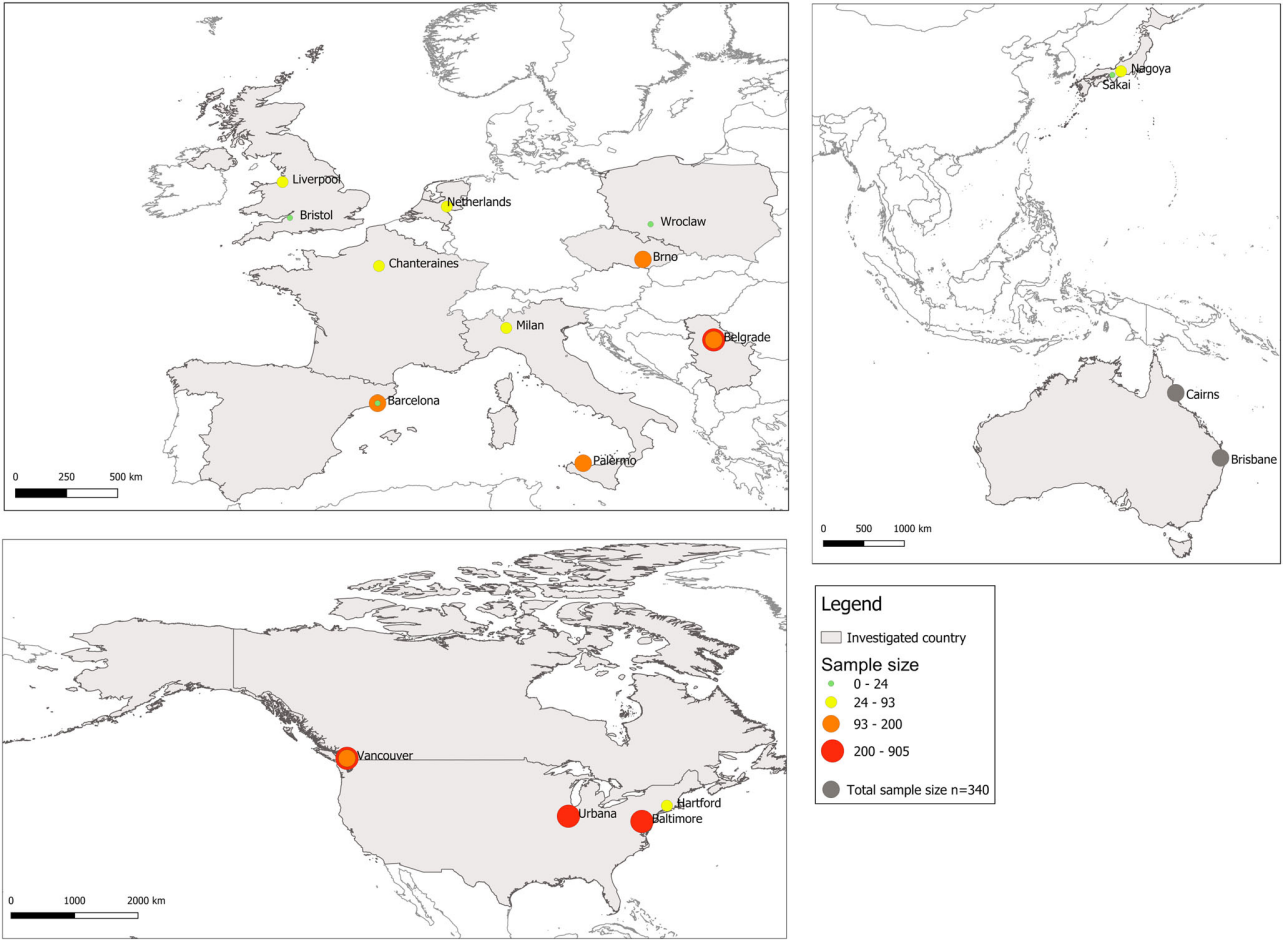
Geographic distribution of the 23 reviewed publications. The size of each circle is proportional to the sample size, i.e. the total number of urban rats (*R. rattus* and *R. norvegicus*) investigated per study

Year of publication of the 23 identified articles ranged between 1946 and 2019, with most of them (*n* = 13, 56.5%) published in the 2010s. Studies were conducted in 12 different developed countries (Fig. 2) on four continents, with the majority of them undertaken in Europe (*n* = 12 papers, 52.2%) and North America (8 papers, 37.8%). Ten papers (43.5%) were published in journals with parasitology as a main scope while five (21.7%) were published in journals showing veterinary medicine as a main scope. The scale of the studies varied greatly: the whole country in one study, two cities in one study, a city in 12 studies, a city and its zoo in two studies, the periurban area of a city in one study, an inner city urban neighbourhood and a port in three studies (those studies belonged to the same project, Vancouver Rat Project: http://www.vancouverratproject.com/vancouver_rat_project/home, and referred to the same sample), a city park in one study and one single animal in two studies. Eleven studies (47.8%) aimed at investigating a single helminth species, two studies aimed at investigating two specific helminth species and nine studies intended to conduct an exhaustive survey of the helminth fauna of urban rats. All studies reported data on urban *R. norvegicus*, while five (21.7%) reported also data on urban *R. rattus.* The median sample size (number of urban rats investigated) was 143 (min. = 1, max. = 905) (Fig. 2).

## Research questions

### Helminths of urban rats

#### Helminth diversity

Helminth taxa from three phyla were reported in urban rats from developed countries, i.e. Acanthocephala, Nematoda and Platyhelminthes, with 25 taxa attributed to the species level (Table 2) and eight to the genus level. *Calodium hepaticum* (syn*. Capillaria hepatica*) was the most commonly reported helminth species in urban rats (14 out of 23 studies), followed by *Heterakis spumosa* (8/23), *Nippostrongylus brasilensis* (7/23), *Hymenolepis diminuta* (the rat tapeworm) (6/23), *Trichosomoides crassicauda* (5/23), *Taenia taeniaformis* (syn. *Strobilocercus fasciolaris*, *Cysticercus fasciolaris*) larvae (4/23), *Syphacia muris* (the rat pinworm) (4/23) and *Rodentolepis* (syn. *Hymenolepis*) *nana* (3/23). The following taxa were recorded in two studies: *Eucoleus gastricus*, *Mastophorus muris*, *Orientostrongylus ezoensis*, *Rodentolepis fraterna*, *Strongyloides ratti*, *Trichuris muris* and *Brachylaima* sp. The following taxa were each recorded in one study: *Angiostrongylus cantonensis* (the rat lungworm), *Aonchotheca annulosa, Aonchotheca murissylvatici*, *Ganguleterakis spumosa*, *Gongylonema neoplasticum*, *Moniliformis moniliformis*, *Notocotylus imbricatus*, *Plagiorchis proximus*, *Rodentolepis microstoma*, *Rodentolepis straminea* and *Strongyloides venezuelensis*. All helminths were retrieved from *R. norvegicus*, among them four were described in *R. rattus*: *A. cantonensis*, *A. murissylvatici*, *C. hepaticum* and *Eucoleus* sp. (Table 2). *Strongyloides venezuelensis* and *O. ezoensis* were reported from Japan only (Yokota et al. 1991; Shintoku et al. 2005).

**Table 2:** Summary of the helminths reported in urban rats (*Rattus norvegicus* and *Rattus rattus*) in cities from developed countries. Those zoonotic are indicated with an asterisk

Helminth	Species authority reported in the reviewed papers	Synonyms found in the reviewed papers	Localisation	Host investigated	Prevalence (%) reported in the reviewed papers (number of studies)
Acanthocephala					
*Acanthocephala* sp.	Not indicated	na	Large intestine	*R. norvegicus*	0.7^a^ (1)
Moniliformis moniliformis*	Not indicated	Thorny-headed worm	Not indicated	*R. norvegicus*	6 (1)
Cestoda					
*Hymenolepis* sp.*	Not indicated	na	Not indicated	*R. norvegicus*	na (1)
Hymenolepis diminuta*	Rudolphi, 1819	Rat tapeworm	Small intestine	*R. norvegicus*	Min.: 1.2–Max. = 36.3 (6)
Rodentolepis microstoma*	Not indicated	na	Small intestine	*R. norvegicus*	8.7 (1)
Rodentolepis nana*	Von Siebold, 1852	Dwarf tapeworm, *Hymenolepis nana*	Small intestine	*R. norvegicus*	Min.: 13.3–Max. = 17.0 (3)
Rodentolepis fraterna*	Stilles, 1906	*Hymenolepis nana* var. *fraterna*	Small intestine	*R. norvegicus*	Min.: 5.3– Max. = 17.8 (2)
Rodentolepis straminea*	Goeze, 1782	na	Not indicated	*R. norvegicus*	40.7 (1)
*Taenia taeniaeformis* larvae	Batsch, 1786	*Cysticercus fasciolaris, Strobilocercus fasciolaris*	Liver	*R. norvegicus*	Min.: 3.7–Max. = 29.3 (4)
Nematoda					
Angiostrongylus cantonensis*	Not indicated	Rat lungworm	Lungs and pulmonary vasculature	*R. norvegicus*	16 (1)
				*R. rattus*	27 (1)
*Aonchotheca annulosa*	Not indicated	na	Not indicated	*R. norvegicus*	12 (1)
*Aonchotheca murissylvatici*	Not indicated	na	Not indicated	*R. norvegicus*	34.2 (1)
			Not indicated	*R. rattus*	50^a^ (1)
*Capillaria* sp.	na	na	Not indicated	*R. norvegicus*	na (2)
*Calodium hepaticum* *	Bancroft, 1893; Moravec, 1982	*Capillaria hepatica*	Liver	*R. norvegicus*	Min.: 10.9–Max. = 94.3^b^ (14)
				*R. rattus*	Min.: 0.0–Max. = 23.7 (2)
*Eucoleus* sp.	na	na	Stomach (non-glandular part) and oesophagus	*R. norvegicus*	na (1)
				*R. rattus*	na (1)
*Eucoleus gastricus*	Not indicated	na	Stomach	*R. norvegicus*	Min.: 28.0–Max. = 30.1 (2)
*Ganguleterakis spumosa*	Not indicated	na	Not indicated	*R. norvegicus*	1.8 (1)
*Gongylonema* sp.	Not indicated	na	Stomach	*R. norvegicus*	na (1)
Gongylonema neoplasticum*	Not indicated	na	Oesophagus	*R. norvegicus*	20 (1)
*Heterakis spumosa*	Schneider, 1866	na	Stomach, lower small intestine, large intestine, caecum, colon	*R. norvegicus*	Min.: 33.3–Max. = 82.5 (8)
*Mastophorus muris*	Gmelin, 1790	na	Stomach	*R. norvegicus*	Min.: 2.4–Max. = 30.6 (2)
*Nippostrongylus brasiliensis*	Travassos, 1914	na	Stomach, upper small intestine, lower small intestine, caecum, colon	*R. norvegicus*	Min.: 6.2–Max. = 100 (7)
*Orientostrongylus ezoensis*	Tada, 1975	na	Stomach, upper small intestine, lower small intestine, large intestine, caecum, colon	*R. norvegicus*	Min.: 88.9–Max. = 94.1 (2)
*Strongyloides* sp.	na	na	Not indicated	*R. norvegicus*	na (1)
*Strongyloides ratti*	Sandground, 1925	na	Stomach, upper small intestine, lower small intestine, large intestine, caecum, colon	*R. norvegicus*	Min.: 11.1–Max. = 97.1 (2)
*Strongyloides venezuelensis*	Not indicated	na	Stomach, upper small intestine, lower small intestine	*R. norvegicus*	75.0 (1)
*Syphacia muris*	Yamaguti, 1935; Yamaguti, 1941	Rat pinworm	Large intestine, caecum	*R. norvegicus*	Min.: 7.0–Max. = 55.0 (4)
*Trichosomoides crassicauda*	Schrank, 1788; Bellingham	na	Urinary bladder, ureters, and kidneys	*R. norvegicus*	Min.: 7.0–Max. = 65.4 (5)
Trichuris muris*	Schrank, 1788	na	Not indicated	*R. norvegicus*	Min.: 2.6–Max. = 8.3 (2)
Trematoda					
*Brachylaima* sp.	na	na	Small intestine	*R. norvegicus*	Min.: 1.2–Max. = 8.4 (2)
*Echinostoma* sp.^c^	na	na	Not indicated	*R. norvegicus*	5.3 (1)
*Notocotylus imbricatus*	Not indicated	na	Not indicated	*R. norvegicus*	10.5 (1)
*Plagiorchis proximus*	Not indicated	na	Not indicated	*R. norvegicus*	7.9 (1)

*na* not applicable. Prevalence is not given when helminths are identified at the genus level only and this genus is reported elsewhere

^a^Sample size = 1

^b^Prevalence calculated on a sample size of one individual excluded

^c^Morphological characteristics were consistent with that of a group of seven *Echinostoma* spp. (*E. chloropodis*, *E. corvi*, *E. hystricosum*, *E. necopinum*, *E. rousseloti*, *E. sarcinum* and *E. travassosi*) (Franssen et al. 2016)

#### Species richness

The helminth species richness per host ranged from one to six (Dyk et al. 1975; Ceruti et al. 2001; Shintoku et al. 2005; Kataranovski et al. 2010; Milazzo et al. 2010; Kataranovski et al. 2011; McGarry et al. 2015; Franssen et al. 2016; Desvars-Larrive et al. 2017; Galan-Puchades et al. 2018).

#### Species association

Within the rat host, a positive association was described between *S. ratti* and *H. spumosa* (Shintoku et al. 2005), *S. muris* and *H. spumosa* (Desvars-Larrive et al. 2017), and *G. neoplasticum* and *M. moniliformis* (Galan-Puchades et al. 2018). The study of Rothenburger et al. (2014b) on urban black and brown rats revealed that, in the host, the presence of *C. hepaticum* in the liver did not impact the presence of *Eucoleus* sp. in the stomach.

#### Prevalence

**Fig. 3:**
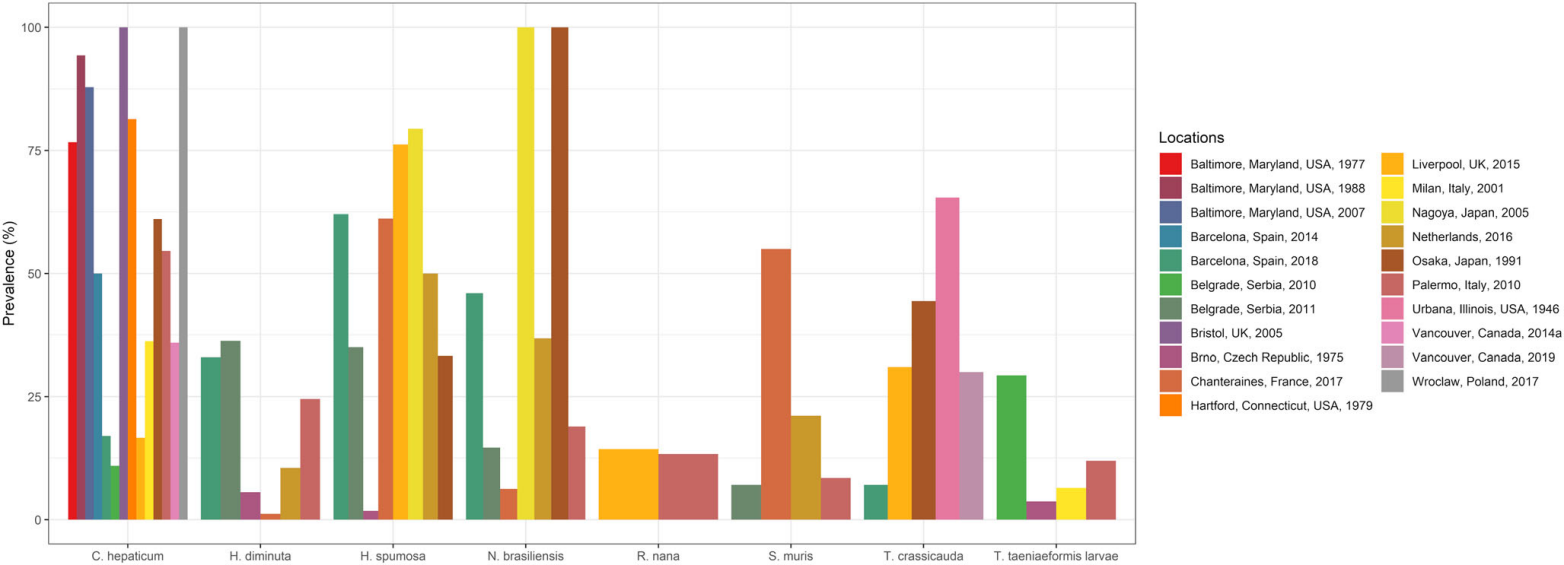
Prevalence of the most common helminth species of the urban brown rat (*R. norvegicus*) in developed countries, as reported in the reviewed papers

Prevalence of each helminth species varied greatly among studies (Table 2) although these prevalences are hardly comparable due to large variations in sample sizes and methods. Figure 3 shows the prevalence of the eight most commonly reported helminth species of the urban brown rat in developed countries.

#### Parasite burden

Parasite burden was reported for the liver larval stage of *T. taeniformis* (Kataranovski et al. 2010), *A. cantonensis* in the lungs (Aghazadeh et al. 2015b), several gastrointestinal helminth species (Yokota et al. 1991; Shintoku et al. 2005; Milazzo et al. 2010; McGarry et al. 2015; Franssen et al. 2016; Desvars-Larrive et al. 2017; Galan-Puchades et al. 2018) and *T. crassicauda* in the urinary tract (Smith 1946). Metrics used to quantify the parasite burden were variable (Supplementary Material 1).

## Methods to investigate the helminths of urban rats

### Identification of the helminths

#### Morphological identification

References to morphological identification keys for the genus or species were given in twelve (52.2%) papers (Yokota et al. 1991; Kataranovski et al. 2010; Milazzo et al. 2010; Kataranovski et al. 2011; Rothenburger et al. 2014a; Rothenburger et al. 2014b; Aghazadeh et al. 2015b; McGarry et al. 2015; Franssen et al. 2016; Bunkowska-Gawlik et al. 2017; Desvars-Larrive et al. 2017; Rothenburger et al. 2019). Drawings and photographs showing typical morphological features of the worms were provided in one (4.3%) (Yokota et al. 1991) and four (17.4%) (Kataranovski et al. 2010; Rothenburger et al. 2014b; Aghazadeh et al. 2015b; Franssen et al. 2016) papers, respectively. We did not consider histological photographs as suitable for morphological identification as they do not show the typical structures to allow species recognition.

Staining procedures for morphological characterisation of the helminths included (i) the use of lactophenol as a clearing solution for the nematodes (Yokota et al. 1991; Galan-Puchades et al. 2018); (ii) the use of Semichon acetocarmine followed by dehydration in alcohol, clearing in xylene and mounting in Canada balsam for the trematodes and cestodes and (iii) the use of Amann lactophenol for temporary mounting of the nematodes and acanthocephalans (Milazzo et al. 2010).

##### Molecular tools

Bunkowska-Gawlik et al. (2017) used a polymerase chain reaction (PCR) to identify *C. hepaticum* eggs retrieved from the liver. A molecular approach was also chosen by Franssen et al. (2016) to distinguish between *H. diminuta*, *R. nana* and *H. fraterna*, but the protocol was not detailed*.*

### Helminths of the liver

Diagnosis of *C. hepaticum* and *T. taeniaformis* larvae was performed through macroscopic examination of the liver to search for typical lesions and confirmed through histological observation of adults and eggs (*C. hepaticum*) or larvae (*T. taeniaformis*) in liver tissue sections. *Calodium hepaticum* eggs cluster unevenly throughout the liver parenchyma, random sectioning is therefore not optimal for the diagnosis (McGarry et al. 2015). *Calodium hepaticum* eggs could also be recovered from liver press preparation (Conlogue et al. 1979; Childs et al. 1988). Quantitative assessment of *C. hepaticum* burden was performed through homogenization of the whole liver, followed by artificial digestion, filtration and count of the total number of eggs under a stereo microscope (McGarry et al. 2015).

### Helminths of the digestive tract

The different segments of the gastrointestinal tract (stomach, upper small intestine, lower small intestine, caecum and colon) were processed separately. Helminths were collected under a stereo microscope after homogenization and filtration of the gastrointestinal content (Franssen et al. 2016) or incubation of the gastrointestinal content in a Baermann apparatus (Shintoku et al. 2005).

Coproscopic investigation was reported in three out of the 23 studies. Samples included fresh faeces collected on the ground (Dyk et al. 1975) and faeco-caecal content (Easterbrook et al. 2007; McGarry et al. 2015). The flotation technique was performed to concentrate eggs for identification of the helminth taxa (Dyk et al. 1975; Easterbrook et al. 2007). A modified McMaster concentration technique was used by McGarry et al. (2015) for egg quantification.

### Helminths of the urinary tract

Presence of *T. crassicauda* was investigated through dissection and observation of the urinary tract under a stereo microscope followed by worm identification using light microscopy (Smith 1946; Yokota et al. 1991; Galan-Puchades et al. 2018) and through histological sections of the urinary tract (McGarry et al. 2015; Rothenburger et al. 2019).

### Helminths of the lungs

*Angiostrongylus cantonensis* was retrieved through the examination of lungs and pulmonary vasculature under a stereo microscope. Identification was confirmed through the observation of male worms under a light microscope (Aghazadeh et al. 2015b).

### Helminths of the muscles

Larvae of *Trichinella spiralis* in the muscle were searched through muscle examination using the compression method (Dyk et al. 1975) or artificial digestion of diaphragm and hind leg muscles followed by sequential filtering (Franssen et al. 2016). Both papers that investigated this parasite reported negative results.

## Risk factors of infection

Seventeen papers (73.9%) investigated potential risk factors of helminth infection in urban brown rats. No risk factors were described for black rats.

### Season

Impact of the season on helminth occurrence varied depending on the geographical location and helminth species. Concerning *C. hepaticum*, the prevalence was stable through the year in Baltimore, USA (Farhang-Azad 1977; Childs et al. 1988; Easterbrook et al. 2007), whereas autumn was at lowest risk of infection in Belgrade, Serbia (Kataranovski et al. 2011), but at highest risk in Vancouver, Canada (Rothenburger et al. 2014a). If the highest prevalences of *H. spumosa*, *N. brasiliensis*, *O. ezoensis*, *S. ratti* and *S. venezuelensis* were reported in spring (March–May) in Japan (Shintoku et al. 2005), the prevalence of *N. brasiliensis* in Europe (Kataranovski et al. 2011) and *H. diminuta* in the USA (Easterbrook et al. 2007) was significantly lower in spring. The prevalence of *Trichuris muris* and *S. muris* was higher in autumn compared to winter in Belgrade, Serbia (Kataranovski et al. 2011). The season did not influence the prevalence of *Eucoleus* sp. in Vancouver, Canada (Rothenburger et al. 2014b). Similarly, the overall prevalence of helminth infection did not show any seasonal variation in Belgrade, Serbia (Kataranovski et al. 2011).

### Host sex

Most of the studies reported no impact of the host sex on the prevalence and parasite burden of helminth infection in urban brown rats (Smith 1946; Farhang-Azad 1977; Childs et al. 1988; Shintoku et al. 2005; Easterbrook et al. 2007; Kataranovski et al. 2010; Milazzo et al. 2010; Rothenburger et al. 2014a; Rothenburger et al. 2014b; McGarry et al. 2015; Desvars-Larrive et al. 2017; Galan-Puchades et al. 2018). However, Kataranovski et al. (2011) reported a higher global prevalence of intestinal helminth infection in male urban brown rats compared to females. Similarly, the prevalence of *C. hepaticum* (Kataranovski et al. 2011), the prevalence of *Hymenolepis* sp. (Easterbrook et al. 2007), the risk of *S. muris* occurrence (Desvars-Larrive et al. 2017), the mean abundance of *M. moniliformis* (Galan-Puchades et al. 2018) and the intensity of infection by *S. ratti* and *S. venezuelensis* (Shintoku et al. 2005) were significantly greater in male urban brown rats compared to females. Rothenburger et al. (2019) evidenced an increased odd of *T. crassicauda* infection in female *R. norvegicus* compared to males.

### Host weight, age and sexual maturity

A positive association was evidenced between host weight and *C. hepaticum* prevalence (Farhang-Azad 1977; Childs et al. 1988; Easterbrook et al. 2007), *Eucoleus* sp. prevalence (Rothenburger et al. 2014b) and *H. diminuta* burden (Galan-Puchades et al. 2018). Smith (1946) and Rothenburger et al. (2019) reported an increased prevalence of *T. crassicauda* in adults and heavier rats (> 145 g), respectively. On the contrary, *Brachylaima* sp. prevalence was reported significantly higher in juveniles than adults (Milazzo et al. 2010) and risk of *S. muris* occurrence was reported significantly lower in heavier rats (Desvars-Larrive et al. 2017). In other studies, no association was evidenced between host weight (or age) and helminth prevalence (Easterbrook et al. 2007; Milazzo et al. 2010; Rothenburger et al. 2014a; Desvars-Larrive et al. 2017; Galan-Puchades et al. 2018). A positive association was found between sexual maturity and odd of infection by *C. hepaticum* (Rothenburger et al. 2014a) and *Eucoleus* sp. (Rothenburger et al. 2014b), independently of the host weight. Pregnancy had no effect on the prevalence of *C. hepaticum* and *Hymenolepis* sp. (Easterbrook et al. 2007).

### Host site of capture

Fine-scale geographic variations in the prevalence of *C. hepaticum* (Farhang-Azad 1977; Childs et al. 1988; Rothenburger et al. 2014a) and *S. muris* (Desvars-Larrive et al. 2017) were reported.

## Pathological findings in urban rats (*R. norvegicus*)

Fourteen (60.8%) of the 23 selected publications reported pathological changes related to the presence of helminths in *R. norvegicus*, i.e. gross lesions and/or histological changes*.*

### Liver

Macroscopic findings associated to *C. hepaticum* consisted in focal, multifocal and sometimes coalescing greyish- or yellowish-white lesions ranging from 1 to 5 mm in diameter on the surface of the organ. The presence of tortuous tracts that resulted from migrating adult *C. hepaticum* was also characteristic (Farhang-Azad 1977; Conlogue et al. 1979; Childs et al. 1988; Ceruti et al. 2001; Easterbrook et al. 2007; Kataranovski et al. 2010; Millán et al. 2014; Rothenburger et al. 2014a; McGarry et al. 2015). Liver lesions were described as slight (i.e. involving a single hepatic lobe) or moderate (i.e. involving less than half of the liver) in the majority of the cases, and, less frequently, as severe (i.e. involving more than half of the liver) (Farhang-Azad 1977; Conlogue et al. 1979; Childs et al. 1988; Ceruti et al. 2001; Kataranovski et al. 2010; Millán et al. 2014). Early stage of infection was characterised by a minor inflammatory process that subsequently evolved into multifocal to coalescent pyogranulomatous hepatitis, progressing to well-defined granulomas surrounding parasitic and necrotic debris. Fibroblastic septa were described as connecting the portal tracts, sometimes also bridging with the terminal hepatic vein. Minor histopathological findings included increase in the diameter of portal and centrolobular veins, hepatic sinusoids containing erythrocytes and a few inflammatory cells, perilobular fibrosis of the liver, hepatocellular necrosis and haemorrhages in the periphery of the lesions and variable stages of biliary hyperplasia.

The presence of *T. taeniaeformis* larvae in the liver was associated with an inflammatory reaction characterised by the presence of periportal eosinophil infiltrates, micro-abscesses, sinusoid enlargement and prominent Kupffer cells (Kataranovski et al. 2010).

### Urinary tract

*Trichosomoides crassicauda* was principally observed in the lumen and superficial mucosa of the urinary bladder (Smith 1946; McGarry et al. 2015; Rothenburger et al. 2019), but also in the lumen and superficial mucosa of the renal pelvis in 30% of the infected rats (Rothenburger et al. 2019) and in the ureters (Smith 1946). Histopathological changes associated with the presence of *T. crassicauda* in the urinary tract consisted in the formation of acellular and mucoid calculi of white to yellowish colour and different shapes in the urinary bladder, the presence of multinucleated cells in the epithelium of the urinary bladder together with a mild epithelial hyperplasia of the urothelium and mild lymphoplasmacytic inflammation of the urinary bladder submucosa (Smith 1946; McGarry et al. 2015; Rothenburger et al. 2019).

### Digestive tract

*Eucoleus* sp. was observed in the non-glandular stomach of *R. norvegicus*. Lesions associated with the presence of this nematode or its eggs included hyperkeratosis, mucosal hyperplasia, keratin pustules and submucosal inflammation (Rothenburger et al. 2014b). A slight hyperaemia of the small intestine was associated with the presence of *R. nana* in (Dyk et al. 1975). On the contrary, McGarry et al. (2015) did not report any pathological changes associated with the presence of *R. nana* or *M. muris*.

## Veterinary and public health importance of rat-borne helminthiases in cities

Sixteen (69.5%) studies highlighted the public health significance of rat-borne helminthiases. Nine potentially zoonotic helminths were mentioned (Table 2): *C. hepaticum* (Farhang-Azad 1977; Conlogue et al. 1979; Childs et al. 1988; Yokota et al. 1991; Ceruti et al. 2001; Easterbrook et al. 2007; Kataranovski et al. 2010; McGarry et al. 2015; Bunkowska-Gawlik et al. 2017; Galan-Puchades et al. 2018), *A. cantonenis* (Aghazadeh et al. 2015a), *G. neoplasticum* (Galan-Puchades et al. 2018), *T. taeniaeformis* (Kataranovski et al. 2010), *R. microstoma* (Desvars-Larrive et al. 2017), *R. nana* (Dyk et al. 1975; McGarry et al. 2015; Galan-Puchades et al. 2018), *H. diminuta* (Easterbrook et al. 2007; Kataranovski et al. 2011; Franssen et al. 2016; Desvars-Larrive et al. 2017; Galan-Puchades et al. 2018), *Brachylaima* sp. (Desvars-Larrive et al. 2017) and *M. moniliformis* (Galan-Puchades et al. 2018). A summary of the main characteristics of these rat-borne helminthiases is provided in Supplementary Material 2.

Seven (30.4%) papers mentioned the potential impact of rat-borne helminthiases on domestic and wild animal health (Conlogue et al. 1979; Childs et al. 1988; Ceruti et al. 2001; Kataranovski et al. 2010; Aghazadeh et al. 2015a; McGarry et al. 2015; Bunkowska-Gawlik et al. 2017). Ceruti et al. (2001) mentioned that only one case of hepatic capillariasis (out of 500 dogs submitted to necropsy over a 10-year period) has been described in domestic carnivores in the study area of Milan, Italy, although the prevalence in urban rats was 36% (unpublished data).

## Discussion

The present systematic review provides an up-to-date overview of the helminths of urban rats in developed countries. Most of the studies were conducted in Europe and investigated brown rats. Twenty-five helminth species were identified at the species level, of which almost two-third belonged to the phylum Nematoda. *Calodium hepaticum* was the most commonly reported helminth in urban rats, mostly because it was the most searched parasite. Some species have a restricted distribution range and were therefore seldom reported. For example, the geographic distribution of *A. cantonensis* is limited to Southeast Asia, the Pacific Islands, South and Central America, and the Caribbean although the parasite is emerging in USA, Australia (Barratt et al. 2016) but also in Europe in the Canary Islands (Martin-Alonso et al. 2011). Similarly, *S. venezuelensis* and *O. ezoensis* are restricted to warmer climates (Viney 2017) and Asia (Fukumoto and Ohbayashi 1985), respectively.

This review underlined that helminth infection is common in wild urban rats and that co-occurrence of multiple helminth species within the same host is frequent. Prevalence, helminth species richness and association, and risk factors of helminth occurrence in urban rats varied greatly between studies. Observed differences may be related to the study design, statistical methods and helminth species investigated. Observed differences may also reflect a high impact of the microenvironment and local climate on the helminth eco-epidemiology.

Pathological findings related to the presence of helminths in their rat hosts were generally minor, except for *C. hepaticum*, which can induce severe hepatic lesions. Positive association between host weight (often, but not always, used as a proxy for age) and prevalence or intensity of helminth infection may suggest that the longer a rat lives, the more likely it is to be parasitized by helminths and the more intense is the infection. The absence of seasonal variation in the prevalence of most helminth species of urban rats probably reflects that transmission occurs year-round. Indeed, warmer temperature and reduced seasonality in cities could enhance the persistence of parasite transmission stages in the environment (Bradley and Altizer 2007). In the near future, climate change and other anthropogenic stressors may induce a loss of parasite biodiversity (Cizauskas et al. 2017) while some parasitic species may adapt and even expand geographically (Blum and Hotez 2018). The distribution range of some geographically restricted helminth species (e.g. *A. cantonensis*, *S. venezuelensis,* and *O. ezoensis*) may be modified due to the expansion of suitable habitat but also introduction events of their intermediate and definitive hosts (York et al. 2014). Therefore, monitoring helminths of urban rats and identifying changes in the parasite-host system may provide insights into the micro-environmental changes occurring within the urban ecosystem.

Urban rats can be definitive, intermediate and sometimes reservoir host of zoonotic helminths (Meerburg et al. 2009; Himsworth et al. 2013). However, the veterinary and public health importance of urban rat helminthiases remains under debate. Symptoms of rat-borne helminthiasis in humans are usually mild or absent, and go unnoticed. Consequently, prevalence of rat-borne helminthiases in the urban population from developed countries as well as environmental sources of contamination remain largely unknown. However, within some cities in the developed countries, poor urban infrastructure and sanitation affect positively rat abundance and increase rat-human contact rate (Byers et al. 2019). People living within impoverished urban areas are therefore more at risk of rat-borne infections, with drug users, immunocompromised and homeless people being the most vulnerable (Delobel et al. 2004; Himsworth et al. 2013; McVea et al. 2018).

This review stresses important limitations regarding research on urban rat helminths. The main limitation lied in the methods of investigation. Field work with urban rats encounters several physical and societal constraints (Desvars-Larrive et al. 2018) which could affect parasitological studies, i.e. place(s) investigated, method(s) of capture, sample size or season of capture. Moreover, helminthological methods are manual and time-consuming; they require a deep and long post-mortem examination of the animal and the collection of each worm individually (or the histological inspection of the liver parenchyma for hepatic helminths). For example, if collection, preservation and staining are straightforward techniques that require minimal equipment, taxonomic identification, especially to species, can be very tedious. Furthermore, helminthology suffers from the lack of consistency in the Latin binomials used to name the species. Discordant use of synonyms for certain helminth species renders literature search and study comparison difficult.

In more than two thirds of the studies, we noticed a dramatic lack of literature references for species identification and absence of morphological description of the reported species. This is particularly necessary for helminths of zoonotic importance, some of which are difficult to readily identify morphologically, i.e. *Moliniformis* spp. (Guerreiro Martins et al. 2017), Hymenolepididae (Casanova et al. 2001; Macnish et al. 2002; Cunningham and Olson 2010; Haukisalmi et al. 2010) and *A. cantonensis* (Aghazadeh et al. 2015a). Overall, the lack of references to the identification keys, the lack of drawings or photographs, the lack of consensus on the taxonomy and names of some helminth species, and the seldom use of molecular tools question the accuracy of the helminth species identification in urban rats.

## Conclusion: future research needs

In order to fill the knowledge gap on the helminths of urban rats, and within the framework of an ethical research, an exhaustive collection and systematic storage of biological material and data related to wild rats, captured (and most of the time euthanized) for scientific purposes, are highly recommended. In this perspective, an extensive inventory of all helminth species from the different organs should be performed for each captured animal. Methods of collection should be standardized as proposed in Sepulveda and Kinsella (2013). Reference to the morphological identification keys of each helminth species is essential. To ensure reproducibility and verification of the study, but also to promote the re-use of the biological material and data for further research, scientists are encouraged, for each helminth species, to (i) deposit a morphological voucher of a male and a female, fixed in formalin (those are then not usable for DNA analysis), in museum collections (Sepulveda and Kinsella 2013); (ii) conserve a voucher specimen in 80–90% ethanol for further DNA analysis. Each voucher specimen should be linked to genetic data deposited in an open-source database (Astrin et al. 2013; Morand 2018), e.g. GenBank (Benson et al. 2012) or BOLD (Ratnasingham and Hebert 2007); (iii) preserve and share raw data (e.g. dataset of morphological data, environmental data, and helminthological data) via open-source repositories.

Helminth species characterisation through traditional DNA sequencing is common, e.g. *H. diminuta* (Julius et al. 2018), *R. straminea*, *R. microstoma*, *R. fraterna* (Macnish et al. 2002; Haukisalmi et al. 2010), *H. spumosa* (Zaleśny et al. 2010; Ribas et al. 2013; Šnábel et al. 2014), *G. neoplasticum* (Setsuda et al. 2018), *M. muris* (Julius et al. 2018), *N. brasiliensis* (Julius et al. 2018) and *S. muris* (Julius et al. 2018). However, facing the limitations of the morphological identification, helminthological studies would highly benefit in the development and use of universal primers for metabarcoding approaches that could screen for multiple parasites concurrently and therefore enable faster, cheaper, more accurate and standardized characterisation of the helminth community within a host species (Tanaka et al. 2014; Aivelo and Medlar 2018).

In addition, the estimation of the parasite burden for each helminth species through count of the individuals, although extremely time-consuming, is essential to the understanding of the ecology of the parasites, in particular to the assessment of potential interspecies interaction within the *Rattus* host. Research on the dynamics and socio-environmental risk factors of human-rat-helminth interaction are missing although they are essential to the understanding of the eco-epidemiology of rat-borne helminthiases in urban ecosystems and to the implementation of surveillance and prevention programmes. Furthermore, few of the reviewed publications stressed the significance of rat-borne helminth infections for other urban animal species. In particular, some helminths of urban rats can infect domestic (dogs and cats) and urban wild carnivores (e.g. foxes) (Štěrba and Baruš 1976; Butcher et al. 1998; Butcher and Grove 2001; Macnish et al. 2003; Jrijer et al. 2015; Mobedi et al. 2017). Finally, there is an important lack of research on the potential use of urban rat helminths as bioindicator for environmental pollution. For example, the host-parasite system rat-*H. diminuta* can be used as a sentinel system to monitor lead contamination in urban ecosystems (Sures et al. 2003). Similarly, *M. moniliformis*, *H. diminuta* and larval stage of *T. taenaeiformis* could be used as bioindicators for assessing environmental pollution by heavy metals (Teimoori et al. 2014).

Overall, we identified several research gaps regarding urban rat helminthology in developed countries, which can be classified into five main topics: (1) species identification through molecular tools, (2) impact of helminth infections on the dynamics of urban rat populations, (3) veterinary and public health importance of rat-borne helminthiases, (4) impact of urbanisation on the helminth populations and (5) potential use of helminths of urban rats as bioindicators for environmental pollution in urban ecosystems.

## Electronic supplementary material

ESM 1 Characteristics of the 23 publications included in the systematic review and extracted data (XLSX 55.1 kb)

ESM 2 Overview of the rat-borne helminthiases mentioned in the 23 reviewed publications and associated reference list (DOCX 62.3 kb)
