# Supplementary material for: Helminths of urban rats in developed countries: a systematic review to identify research gaps
Source: Parasitol Res. 2020 Jun 30;119(8):2383–97. doi: 10.1007/s00436-020-06776-3 (PMC7366588; doi:10.1007/s00436-020-06776-3)
Supplement: Supplementary file 2 — Overview of the rat-borne helminthiases mentioned in the 23 reviewed publications and associated reference list (DOCX 62.3 kb) [file 436_2020_6776_MOESM2_ESM.docx]

**Supplementary Material 2**

**Helminths of urban rats in developed countries: A systematic review to identify research gaps**

**Authors:** Diana S. Gliga, Benoît Pisanu, Chris Walzer, Amélie Desvars-Larrive

**Corresponding author:** Amélie Desvars-Larrive

**Corresponding author´s affiliations:**

1 Conservation Medicine, Research Institute of Wildlife Ecology, University of Veterinary Medicine Vienna, Austria

2 Unit of Veterinary Public Health and Epidemiology, Institute of Food Safety, Food Technology and Veterinary Public Health, University of Veterinary Medicine Vienna, Austria

3 Complexity Science Hub Vienna, Austria

**Corresponding author´s email:** [amelie.desvars@vetmeduni.ac.at](mailto:amelie.desvars@vetmeduni.ac.at)

**Supplementary Material 2** Overview of the rat-borne helminthiases mentioned in the 23 reviewed publications and associated reference list

| **Helminth species** | **Hosts** | **Mode of transmission to humans** | **Symptoms in humans** | **Occurrence in humans** |
| --- | --- | --- | --- | --- |
| *Angiostrongylus cantonenis* | Definitive hosts: rats (*Rattus* spp.).  Accidental hosts: humans, domestic carnivores, wildlife and birds.  Intermediate hosts: gastropods (molluscs). | Consumption of raw or undercooked intermediate hosts (Aghazadeh et al. 2015; Centers for Disease Control and Prevention 2019c). | Symptoms range from mild (i.e. flu-like) to severe (i.e. eosinophilic meningitis, paralysis, coma), rarely, the disease could be fatal (Chaisiri et al. 2015). | 2,827 cases of the disease have been documented worldwide. Children are more at risk of infection (Chaisiri et al. 2015; Centers for Disease Control and Prevention 2019c). |
| *Brachylaima* sp. | First and second intermediate hosts (carry the sporocysts and metacercariae stages): land snails (Gállego et al. 2014).  Definitive hosts: mammals (including humans) and birds | Main route: accidental ingestion of infected snails carrying the metacercariae.  Minor route: consumption of raw or undercooked snails (Butcher et al. 1998; Butcher and Grove 2001; Butcher 2016). | Often asymptomatic. Clinical symptoms include abdominal pain, intermittent diarrhoea, anorexia, vomiting (Butcher et al. 1996; Butcher et al. 1998). | 15 laboratory-confirmed human infections by *Brachylaima cribbi* (Australia) (Butcher 2016). Children < 2 years are more at risk. |
| *Calodium hepaticum* | Definitive hosts: several wild and domestic urban mammals including micromammals (usually rodents), but also lagomorphs, dogs, cats, and wild carnivores (e.g. foxes) (Spratt and Singleton 2008). | Ingestion of embryonated eggs. | Usually asymptomatic but can sometimes induce hepatitis, anemia, fever, hypereosinophilia, and in rare cases may be fatal. The larvae can occasionally migrate to the lungs, kidneys, or other organs (Centers for Disease Control and Prevention 2017a). | 163 cases recorded worldwide in the literature (Fuehrer 2014). |
| *Gongylonema neoplasticum* | Intermediate hosts: coprophagous arthropods. | Ingestion of infective larvae stage carried by an intermediate host (Jrijer et al. 2015). | Whether *G.* *neoplasticum* infection leads to symptoms in humans is still controversial (Jrijer et al. 2015). Patients describe a sensation of an object moving in the mouth (Wilson et al. 2001). | 40–50 cases of infection by *Gongylonema* sp. have been reported in humans (Europe, North Africa, China, New Zealand, Sri Lanka and the USA) (Wilson et al. 2001). |
| *Hymenolepis diminuta* | Reservoir hosts: rodents.  Accidental hosts: humans (Centers for Disease Control and Prevention 2017b). | Accidental ingestion of the cysticercoid-infected intermediate arthropod hosts (Centers for Disease Control and Prevention 2017b). | Generally no severe symptoms (Butcher and Grove 2001). Symptoms can include abdominal pain, irritability, and pruritus (Tena et al. 1998). | About 500 cases of H. diminuta in humans have been sporadically reported worldwide (Tena et al. 1998). Children are more at risk of infection (Ahmad et al. 2017). |
| *Moniliformis moniliformis* | Definitive hosts: rodents, dogs, foxes, and cats, rarely humans.  Intermediate hosts: arthropods (mostly beetles or cockroaches) (Berenji et al. 2007). | Ingestion of infective larvae stage carried by an intermediate host (Centers for Disease Control and Prevention 2019b). | Can be asymptomatic. Can induce abdominal pain, diarrhoea, vomiting, irritability, weakness (Counselman et al. 1989; Berenji et al. 2007; Messina et al. 2011). | Scarcely described (cases have been reported from Australia, Iran, Iraq, Nigeria, USA) (Chaisiri et al. 2015). |
| *Rodentolepis microstoma* | Definitive hosts: mice, gerbils, voles, hamsters (Macnish et al. 2003; Cunningham and Olson 2010). Infection in rats is controversial (Cunningham and Olson 2010).  Intermediate hosts: beetles (Cunningham and Olson 2010). | Main route: ingestion of the arthropod intermediate host Minor route: direct human-to-human transmission (Macnish et al. 2003). | Zoonotic potential uncertain (Macnish et al. 2003). | One report in humans (Australia) (Macnish et al. 2003). |
| *Rodentolepis nana* | Definitive hosts: humans (Centers for Disease Control and Prevention 2019a).  Intermediate hosts (facultative in the life cycle): arthropods (mainly fleas and beetles). | Main route: direct ingestion of eggs.  Minor route: ingestion of the facultative arthropod intermediate host (Centers for Disease Control and Prevention 2017b).  Other: auto-infection which can persist for years (Štěrba and Baruš 1976). | Rarely causes symptoms (Centers for Disease Control and Prevention 2017b). | Responsible for the majority of human tapeworm infections, with an estimated number of 50,000,000-75,000,000 carriers worldwide. Most often seen in children in countries with poor sanitation and hygiene (Centers for Disease Control and Prevention 2019a). |
| *Taenia taeniaeformis* | Definitive hosts (carry *T. taeniformis* in the intestine): carnivores of the families *Felidae*, *Canidae,* and *Mustelidae*, including domestic cats and dogs (Chaisiri et al. 2015)*.*  Intermediate hosts (carry the larval stage, *C. fasciolaris*, in the liver): rodents (Lee et al. 2016) but also humans (Štěrba and Baruš 1976; Hoberg 2002). | Consumption of food infected with cat faeces (Ekanayake et al. 1999). | Rarely causes symptoms. | Number of reported cases of *T. taeniaeformis* larvae in humans is low (Štěrba and Baruš 1976; Hoberg 2002). |

^.a^ Number of documented cases, if available.

**References**

Aghazadeh M, Reid SA, Aland KV, Restrepo AC, Traub RJ, McCarthy JS, Jones MK (2015) A survey of *Angiostrongylus* species in definitive hosts in Queensland. Int J Parasitol Parasites Wildl 4:323-328. <https://doi.org/10.1016/j.ijppaw.2015.06.003>

Ahmad AF, Ngui R, Ong J, Sarip F, Ismail WHW, Omar H, Nor ZM, Amir A, Lim YAL, Mahmud R (2017) Case report: a symptomatic case of *Hymenolepis diminuta* infection in an urban-dwelling adult in Malaysia. Am J Trop Med Hyg 97:163-165. <https://doi.org/10.4269/ajtmh.15-0877>

Berenji F, Fata A, Hosseininejad Z (2007) A case of *Moniliformis moniliformis* (Acanthocephala) infection in Iran. Korean J Parasitol 45:145-148. <https://doi.org/10.3347/kjp.2007.45.2.145>

Butcher A (2016) Children, snails and worms: the *Brachylaima cribbi* story. Microbiol Aust 37:30-33. 10.1071/MA16012

Butcher AR, Grove DI (2001) Description of the life-cycle stages of *Brachylaima cribbi* n. sp. (Digenea: *Brachylaimidae*) derived from eggs recovered from human faeces in Australia. Syst Parasitol 49:211-221. <https://doi.org/10.1023/A:1010616920412>

Butcher AR, Parasuramar P, Thompson CS, Grove DI (1998) First report of the isolation of an adult worm of the genus *Brachylaima* (Digenea: *Brachylaimidae*), from the gastrointestinal tract of a human. Int J Parasitol 28:607-610. <https://doi.org/10.1016/S0020-7519(97)84372-X>

Butcher AR, Talbot GA, Norton RE, Kirk MD, Cribb TH, Forsyth JRL, Knight B, Cameron AS (1996) Locally acquired *Brachylaima* sp. (Digenea: Brachylaimidae) intestinal fluke infection in two South Australian infants. Med J Australia 164:475-478. <https://doi.org/10.5694/j.1326-5377.1996.tb122125.x>

Centers for Disease Control and Prevention (2017a) DPDx - Laboratory Identification of Parasites of Public Health Concern - Hepatic Capillariasis. <https://www.cdc.gov/dpdx/hepaticcapillariasis/index.html>. Accessed 06/08/2019 2019

Centers for Disease Control and Prevention (2017b) DPDx - Laboratory Identification of Parasites of Public Health Concern - Hymenolepiasis. <https://www.cdc.gov/dpdx/hymenolepiasis/index.html>. Accessed 06/08/2019 2019

Centers for Disease Control and Prevention (2019a) DPDx - Laboratory Identification of Parasites of Public Health Concern. <https://www.cdc.gov/dpdx/acanthocephaliasis/index.html>. Accessed 07/08/2019 2019

Centers for Disease Control and Prevention (2019b) DPDx - Laboratory Identification of Parasites of Public Health Concern - Acanthocephaliasis. <https://www.cdc.gov/dpdx/acanthocephaliasis/index.html>. Accessed 07/08/2019 2019

Centers for Disease Control and Prevention (2019c) Parasites - Angiostrongyliasis (also known as Angiostrongylus Infection). <https://www.cdc.gov/parasites/angiostrongylus/index.html> Accessed 06/08/2019 2019

Chaisiri K, Siribat P, Ribas A, Morand S (2015) Potentially zoonotic helminthiases of murid rodents from the Indo-Chinese peninsula: impact of habitat and the risk of human infection. Vector Borne Zoonotic Dis 15:73-85. <https://doi.org/10.1089/vbz.2014.1619>

Counselman K, Field C, Lea G, Nickol B, Neafie R (1989) *Moniliformis moniliformis* from a child in Florida. Am J Trop Med Hyg 41:88-90. <https://doi.org/10.4269/ajtmh.1989.41.88>

Cunningham LJ, Olson PD (2010) Description of *Hymenolepis microstoma* (Nottingham strain): a classical tapeworm model for research in the genomic era. Parasit Vectors 3:123-123. <https://doi.org/10.1186/1756-3305-3-123>

Ekanayake S, Warnasuriya ND, Samarakoon PS, Abewickrama H, Kuruppuarachchi ND, Dissanaike AS (1999) An unusual ‘infection’ of a child in Sri Lanka, with Taenia taeniaeformis of the cat. Annals of Tropical Medicine & Parasitology 93:869-873. <https://doi.org/10.1080/00034983.1999.11813494>

Fuehrer H-P (2014) An overview of the host spectrum and distribution of *Calodium hepaticum* (syn. *Capillaria hepatica*): part 1—Muroidea. Parasitol Res 113:619-640. <https://doi.org/10.1007/s00436-013-3691-x>

Gállego L, González-Moreno O, Gracenea M (2014) Terrestrial edible land snails as vectors for geographic dissemination of *Brachylaima* species. J Parasitol 100:674-678, 675. <https://doi.org/10.1645/13-386.1>

Hoberg EP (2002) *Taenia* tapeworms: their biology, evolution and socioeconomic significance. Microbes and Infection 4:859-866. <https://doi.org/10.1016/S1286-4579(02)01606-4>

Jrijer J, Bordes F, Morand S, Neifar L (2015) A survey of nematode parasites of small mammals in Tunisia, North Africa: diversity of species and zoonotic implications. Comp Parasitol 82:204-210, 207. <https://doi.org/10.1654/4767.1>

Lee B-W, Jeon B-S, Kim H-S, Kim H-C, Yoon B-I (2016) *Cysticercus fasciolaris* infection in wild rats (R*attus norvegicus*) in Korea and formation of cysts by remodeling of collagen fibers. J Vet Diagn Invest 28:263-270. <https://doi.org/10.1177/1040638716643129>

Macnish MG, Ryan UM, Behnke JM, Thompson RCA (2003) Detection of the rodent tapeworm Rodentolepis (=Hymenolepis) microstoma in humans. A new zoonosis? Int J Parasitol 33:1079-1085. <https://doi.org/10.1016/S0020-7519(03)00137-1>

Messina AF, Wehle FJJ, Intravichit S, Washington K (2011) *Moniliformis moniliformis* infection in two Florida toddlers. Pediatr Infect Dis J 30:726-727. 10.1097/INF.0b013e31821e52e9

Spratt D, Singleton G (2008) Hepatic Capillariasis. In: Samuel WM, M. Pybus, and A. Kocan (ed) Parasitic Diseases of Wild Mammals. Manson Publishing/The Veterinary Press, London, UK, pp 365-379. <https://doi.org/10.1002/9780470377000.ch14>

Štěrba J, Baruš V (1976) First record of *Strobilocercus fasciolaris* (*Taeniidae* - larvae) in man. Folia Parasitol 23:221-226

Tena D, Pérez Simón M, Gimeno C, Pérez Pomata MT, Illescas S, Amondarain I, González A, Domínguez J, Bisquert J (1998) Human infection with *Hymenolepis diminuta*: case report from Spain. J Clin Microbiol 36:2375-2376

Wilson ME, Lorente CA, Allen JE, Eberhard ML (2001) *Gongylonema* infection of the mouth in a resident of Cambridge, Massachusetts. Clin Infect Dis 32:1378-1380. <https://doi.org/10.1086/319991>
